# Marketing claims on the websites of leading e-cigarette brands in England

**DOI:** 10.1136/tc-2023-057934

**Published:** 2023-07-04

**Authors:** Matilda Kim Nottage, Eve Violet Taylor, Yebin Kim, Nicole Soh, David Hammond, Erikas Simonavicius, Ann McNeill, Deborah Arnott, Katherine East

**Affiliations:** 1 Addictions Department, King's College London, London, UK; 2 School of Public Health and Health Systems, University of Waterloo, Waterloo, Ontario, Canada; 3 Shaping Public Health Policies to Reduce Inequalities and Harm (SPECTRUM) Consortium, Edinburgh, UK; 4 Action on Smoking and Health, London, UK

**Keywords:** Electronic nicotine delivery devices, Public policy, Nicotine, Advertising and Promotion

## Abstract

**ABSTRACT:**

**Introduction:**

Exposure to electronic cigarette (EC) marketing is associated with EC use, particularly among youth. In England, the Tobacco and Related Products Regulations and Committee of Advertising Practice (CAP) regulate EC marketing to reduce appeal to youth; however, there are little published data on EC marketing claims used online. This study therefore provides an overview of marketing claims present on the websites of EC brands popular in England.

**Methods:**

From January to February 2022, a content analysis of 10 of England’s most popular EC brand websites was conducted, including violation of CAP codes.

**Results:**

Of the 10 websites, all presented ECs as an alternative to smoking, 8 as a smoking cessation aid and 6 as less harmful than smoking. Four websites presented ECs as risk-free. All mentioned product quality, modernity, convenience, sensory experiences and vendor promotions. Nine featured claims about flavours, colours, customisability and nicotine salts. Seven featured claims concerning social benefits, personal identity, sustainability, secondhand smoke and nicotine strength. Six featured claims about fire safety. Some claimed ECs are cheaper than tobacco (n=5), cited health professionals (n=4) or featured collaborations with brands/icons (n=4). All were assessed by the research team to violate one or more CAP code(s) by featuring medicinal claims (n=8), contents which may appeal to non-smokers (n=7), associations with youth culture (n=6), depictions of youth using ECs (n=6) or media targeting youth (n=5).

**Conclusion:**

Among 10 top EC brand websites in England, marketing elements that might appeal to youth were commonly identified and CAP code compliance was low.

**WHAT IS ALREADY KNOWN ON THIS TOPIC**

- Online marketing of electronic cigarette (EC) products is increasingly noticed by youth in England and may affect EC uptake. No studies have investigated the marketing content present specifically on EC manufacturers’ own websites.

**WHAT THIS STUDY ADDS**

- The EC market is fast-changing, as is the body of evidence on EC harms and benefits and subsequently the regulatory framework. This study offers an overview of marketing claims used on the websites of commonly used EC brands in England in 2022 and assesses compliance with current regulations.

**HOW THIS STUDY MIGHT AFFECT RESEARCH, PRACTICE OR POLICY**

- Our findings highlight the need for more explicitly worded and better enforced regulations updated to reflect recent evidence on EC use for smoking cessation.

## Introduction

 Electronic cigarette (EC) use is widespread in England, with an estimated 4.3 million adult vapers in 2022.^1^ ECs carry fewer health risks than combustible tobacco and may support smoking cessation^1^,^5^; the National Institute for Health and Care Excellence offers guidance on the use of nicotine-containing ECs for smoking cessation. However, ECs are also gaining popularity among youth in the UK, with self-reported ever EC use increasing from 11.2% to 15.8% between 2021 and 2022 and current use from 3.3% to 7.0% (3.1% occasional and 3.9% regular) over this same period among those aged 11–17 years old.^6^ This increase among youth is concerning because ECs are not risk-free, long-term health consequences are yet unknown and ECs can contain nicotine which is addictive.^1 5^ While use among non-smoking youth is for the most part experimental,^6^ it is important to ensure that EC uptake among youth and non-smokers remains minimal.

Marketing and advertising may play an important role in the uptake of ECs among youth and non-smokers. Youth exposure to EC advertising in England is frequent and has increased since 2017,^7^,^9^ which is concerning because exposure to EC marketing has been associated with EC trial and past-month use among youth.^9^,^11^ This is consistent with the combustible tobacco literature which shows that marketing can influence cigarette appeal and use.^12 13^ EC marketing through online channels is especially relevant when considering youth uptake because youth report noticing online EC marketing to a greater extent than adults: 41.1% of youth (aged <18 years) reported noticing EC marketing through websites or social media in the UK in 2018, compared with 13.3% of adults aged 25 years and over.^14^ Furthermore, exposure to online EC marketing is associated with regular EC use among youth.^15^

It should be noted that most studies investigating the effects of EC marketing exposure online do not differentiate between websites and social media,^8 10 15^ and that youth may not be exposed to websites as much as they are exposed to social media. The EC marketing content used on social media has been the subject of more research than EC brands’ websites.^16 17^ These websites should be investigated as they provide an overview of the marketing contents used by popular brands across various online media.

Furthermore, EC retailer websites are one of the main legal channels for online EC marketing in England. The Tobacco and Related Products Regulations (TRPR) regulate EC products and prohibit EC marketing through certain media channels^18^; non-broadcast advertising, which includes websites, is monitored and regulated by the Advertising Standards Authority’s (ASA) Committee of Advertising Practice (CAP). CAP codes prohibit advertising though certain online media, reflecting the TRPR, but do not prohibit EC marketers from using ‘factual claims about products’ on their own websites.^19 20^ Specifically, CAP codes 22.1–22.12 are intended to protect non-smokers and youth from exposure to non-broadcast EC marketing and EC uptake.^19^ They prohibit certain content including association with/promotion of tobacco products, medicinal claims (including smoking cessation), endorsements by health professionals, displaying youth using ECs, reflecting youth culture or using media directed at youth.

Numerous EC websites can be accessed in England: most are extensive, update their contents frequently and the extent to which they comply with regulations is unclear. Regulating online marketing is made difficult by the international nature of the internet; the self-reported frequency of youth exposure to EC marketing via websites or social media has been found to be similar across England, Canada and the USA, despite different marketing regulations.^7^ Considering the attention paid to online EC marketing by youth,^14^ an assessment of the marketing strategies used with EC brands online will identify potential influences on youth and never-smokers. In particular, manufacturers’ own websites might employ unique marketing strategies to encourage brand loyalty.

Content analyses of EC retail websites have been conducted in China and the USA^21 22^ and more recently in New Zealand and Australia.^23 24^ These analyses revealed the common use of claims concerning health benefits of ECs and their potential for smoking cessation, and many highlighted the wide variety of flavours available for sale. In England, analyses of marketing content and compliance with regulations have been conducted for EC marketing in traditional and social media.^25^ However, no studies to date have focused on EC manufacturers’ own websites. This study aims to provide an overview of the claims which can be found on the websites of EC brands popular in England in 2022, considering compliance with current regulations.

## Methods

### Sample

Websites were selected based on brand popularity as reported by youth and adult past 30-day vapers through the International Tobacco Control (ITC) Youth Tobacco and Vaping Survey (September 2021; aged 16–19 years in England) and the ITC Four Country Smoking and Vaping Survey (September 2020; aged 18+ years in England). We selected the top 10 most used brands among youth and top 10 among adults. Of these, eight overlapped and two were excluded because their websites were third-party retailers (not brand specific), leaving 10 unique brands. Other brands were reported by very few respondents, reflecting data that the EC market share is typically dominated by a select few brands at one time point.^26^ The 10 brand websites analysed were: 88Vape, Aspire, Blu, Innokin, JUUL, Logic, Smok, Vaporesso, Voopoo and Vuse (formerly ‘Vype’).

### Procedure

We developed a codebook of the key characteristics of EC retail websites and EC marketing claims based on previous scans,^21 27^ a review of UK regulations on EC advertising^18 19^ and a pilot scan of 4 of the 10 selected websites. Table 1 provides an overview of the codebook categories. The complete codebook is in online supplemental appendix 1. Of the 12 CAP codes, 10 were included in the codebook (codes 22.2–22.11), one was analysed separately based on the identified marketing claims (code 22.12: ‘Factual claims about products are permitted on marketers’ own websites’) and one was excluded because it could not be operationalised (code 22.1: ‘Marketing communications for e-cigarettes must be socially responsible’).

**Table 1:** Overview of codebook categories

Codebook categories	Description
Website description	Date of access, URL, country specificity, age restrictions
Product availability	Types of EC devices and refills sold: open tank devices, cartridge devices, ‘cigalike’ disposable devices, e-liquid bottles and cartridges
Marketing claims	(1) Comparisons with tobacco and health-related claims (including smoking cessation claims), (2) claims relating to product characteristics, (3) financial claims, (4) social claims, (5) claims relating to flavour, nicotine and sensory experiences, (6) claims relating to modernity and convenience, and (7) claims relating to collaborations
CAP code compliance	Compliance with each of CAP codes 22.2–22.11

CAP, Committee of Advertising Practice; EC, electronic cigarette; URL, Uniform Resource Locator.

Websites were accessed between 13 January and 21 February 2022. Each website was double-coded; all pages and features (eg, images and videos) were considered. Codes were not mutually exclusive. Screenshots provided a record of claims’ appearance on websites at the time of data collection. Coders wrote justifications for their assessment of claims and of CAP code compliance. Some CAP codes were vague, requiring coders to make subjective decisions about whether claims violated them. A third coder subsequently settled any inconsistencies, with discussions among the team where claims were unclear. Following data collection, we coded whether the claims that were extracted from the websites were factual, in line with code 22.12 (‘Factual claims about products are permitted on marketers’ own websites’; referring to the specifications available on the CAP’s website^20^).

## Results

### Website and product characteristics

Among the 10 selected EC brand websites, 5 had a UK-specific domain (eg, ‘.co.uk’ or ‘/gb’) and 2 did not offer direct retail. All but one (n=9) required users to click to confirm that they were of legal age to purchase EC products in the UK (18 years or over) before accessing the website, but none had age verification procedures in place to restrict website access. All brands sold at least one EC device that worked with e-liquid cartridges (four using refillable cartridges, three using disposable cartridges, three using either). Nine brands sold at least one device with an open tank which can be refilled, and one brand sold disposable ‘cigalike’ devices. Six brands sold e-liquid refills (bottles and/or prefilled cartridges).

### Marketing claims

Table 2 shows the marketing claims that were assessed and the frequency with which they appeared across the 10 websites.

**Table 2:** Appearance of marketing claims across the top 10 EC brand websites in England

Marketing claims	Frequency	Example
Comparisons with tobacco and health-related claims		
ECs as an alternative to smoking	10	‘Intended for adult smokers seeking a satisfying alternative to cigarettes’
ECs as less harmful than smoking	6	‘Clear scientific evidence and the support of governments that vaping ECs is less harmful than smoking’
ECs as a smoking cessation aid	8	‘These devices will help you quit smoking’
ECs as risk-free or harmless	4	‘You can vape safely knowing that there are no harmful ingredients’
Claims relating to product characteristics		
Product quality	10	‘We use only high-quality ingredients, conduct rigorous testing, design our products with safety features’
Personalisation or customisation of the product(s)	9	‘Keep it sleek and sophisticated with a discreet black carry case, or find one emblazoned with your favourite Marvel character.’
Availability of different colours	9	‘Available in 6 different color options to match your style’
Sustainability or ‘eco-friendliness’ of the product(s)	7	‘We are taking steps to deliver more sustainable vaping products to you and reduce our impact on the environment. Join us. Together we can be kinder to the planet.’
Fire safety of the product(s)	6	‘A range of built-in safety protections, courtesy of the ASP chipset, including overcharge, overheat and short circuit, delivers a secure and long-lasting vaping experience.’
Financial claims		
Vendor promotions or offers	10	‘Share your stories with us and win a [product)! Comment below about your personal vaping experience’
Cheaper cost compared with tobacco	5	‘A cost that is 80% lower than buying a packet of cigarettes’
Social claims		
Reference to an increased ability to socialise	7	‘People will avoid smokers because they hate the odour(…)when you switch from smoking to vaping, you'll notice that people will want to spend more time with you.’
ECs not bothering non-smokers or lack of secondhand smoke	7	‘Vaping doesn't release any irritating substance that could cause disturbance to the people around.’
Association of ECs with personal identity	7	‘Brand new designs will help show off your style and personality.’
Claims relating to flavour, nicotine and sensory experiences		
Sensory experiences associated with ECs	10	‘Providing a smooth and flavorful mouth to lung vape with all the throat hit you may need.’
Availability of different flavours	9	‘There is a taste for everyone with over 50 delicious e liquid flavours to choose from’
Availability of nicotine salts	9	‘Nicotine salts offer a richer vapour, for a more intense vaping experience.’
Reference to nicotine strength	7	‘[brand] offers different strength and flavour options to help you find your satisfaction.’
Claims relating to modernity and convenience		
EC products as ‘modern’ or ‘innovative’	10	‘Modernising the traditional EC with a sleek design’
Convenience of use	10	‘Designed to be convenient, easy-to-use, and satisfying’
Claims relating to collaborations		
Collaborations with celebrities, musicians, artists, brands or other icons	4	‘Whether it’s big concerts, small jams, art exhibitions, comedy or sports events, you can find [brand] products at the following venues across the UK.’ (logos of clubs and venues)

EC, electronic cigarette.

#### Comparisons with tobacco and health-related claims

All websites presented EC products as a smoking alternative. Eight mentioned the potential of ECs as a smoking cessation aid; one mentioned that ‘Whilst vaping is not licensed by MHRA [Medicines and Healthcare products Regulatory Agency] as a cessation product yet, thousands of people in the UK have stopped smoking with the help of an e-cigarette’, and another brand stated that their mission is to ‘transition the world’s billion adult smokers away from combustible cigarettes’. Four brands used health professionals or health bodies to endorse the benefits of EC products, for instance quoting a Professor of Medicine about the relative harm of ECs. Six websites claimed that ECs are safer than combustible tobacco, with one brand specifically claiming that their devices contained ‘99% fewer toxicants than cigarettes’. Four brands featured claims about ECs being risk-free or harmless.

#### Claims relating to product characteristics

All brands featured positive claims about the quality of their products, mentioning the device’s build, the manufacturing process and e-liquid ingredients. Six brands referred to the fire safety of their devices. Environmental sustainability claims (n=7) ranged from prominent (eg, a dedicated sustainability page) to brief (eg, recycling icons). Some featured claims about being carbon neutral, and one website boasted a large green banner on its homepage, advertising the recycling of disposables, calling to ‘Protect the Earth’, and promising they would ‘treat waste in a harmless manner’. Nine websites featured claims about customisability of EC products, including advertising devices with varying wattage for lighter or stronger vapour. Customisability also referred to aesthetic elements, for example, offering ‘distinctive colors to match your style’. Indeed, colour was a widespread personalisation tool, with all but one website (n=9) offering a range of colour options; engraving services were also offered on some websites.

#### Financial claims

All websites featured some form of vendor promotion, such as limited-time discounts, price reductions when buying multiple products and games with EC products as prizes. Five websites claimed that the EC products they sold were cheaper than tobacco products. One brand used the chance of winning EC products as an incentive to encourage users to share testimonials about their vaping experiences.

#### Social claims

Seven brands claimed that the use of their EC products could confer an increased ability to socialise. One subtype of social claims concerned reduced stigma compared with smoking. For instance, one website included testimonials of users saying they did not feel stigmatised when using ECs, and another claimed that users would have more professional success once they stopped ‘smelling like an ashtray’. There was significant overlap between these claims and those advertising the lack of harmful or bothersome secondhand smoke from ECs compared with combustible tobacco (n=7). A second subtype of social claims concerned social benefits, focusing on using ECs to connect with a new community and develop a social identity as a vaper. For instance, one brand launched a social platform to ‘make friends with other vapers all over the world’. Similarly, seven brands directly related product characteristics to personal identity.

#### Flavour, nicotine strength and associated sensory experiences

In addition to customisability and colours, many websites offered a choice of flavours (n=9) and nicotine content (n=7). Flavour options were often varied and described using promotional terms such as ‘delicious’. The choice of nicotine strength was often framed as a way of customising the product according to preference and experience. Products containing nicotine salts were available on nine websites and were at times described in relation to sensory experiences. In fact, all websites included references to positive sensory experiences associated with EC use: common claims referred to flavour, the quality and/or amount of vapour, or the ‘throat hit’ associated with nicotine-containing products. One website advertised the benefits of enjoying sweet EC flavours ‘without worrying about the calories’.

#### Claims relating to modernity and convenience

Claims about modernity and innovation were found on all websites. Some focused on the modernity of a specific product or design, while others used broader claims. For instance, one website featuring a video of a group of young people taking a ‘selfie’ overlaid with text stating: ‘Innovation keeps changing the vaping experience’. Claims relating to convenience were also identified among every website. Most focused on ease of use, for example, ‘It’s compact, easy to use and hassle free’, some advertised ECs as ‘discreet’ and one drew a comparison with smoking, stating: ‘It’s easier to vape than smoke in some public places’.

#### Claims relating to collaborations

Four websites featured collaborations with ‘icons’, such as artists, celebrities or brands. One brand had collaborations with street artists. Another featured a page dedicated to collaborations with events and venues, including the logos of popular nightclubs, concerts and sports events.

### CAP code compliance

An overview of our assessment of compliance with CAP codes 22.2–22.11 can be found in table 3. We assessed all websites to violate at least one code, with three violating one or two code(s) and seven websites violating between four and six codes. Eight websites featured claims about smoking cessation, which are considered medicinal by the ASA and thus prohibited by code 22.5.^28^ Other frequently violated codes concerned appeal to non-smokers (code 22.8, n=7), appeal to youth (aged <18 years) (code 22.9, n=6) and depictions of people under 25 years using ECs (code 22.10, n=6).

**Table 3:** Compliance with CAP codes 22.2–22.11 across the top 10 EC brand websites in England

CAP code	Frequency of websites that violated codes	Example
22.5 Marketing communications **must not contain medicinal claims** unless the product is authorised for those purposes by the MHRA. E-cigarettes may be presented as an alternative to tobacco but marketers must do nothing to undermine the message that quitting tobacco use is the best option for health.*	8	*‘*These devices will help you quit smoking.’
22.8 Marketing communications **must not encourage non-smokers or non-nicotine-users** to use e-cigarettes.	7	‘As a popular brand, we’ve got a range of [brand] starter kits for beginners and experienced vapers’
22.9 Marketing communications **must not be likely to appeal particularly to people under 18, especially by reflecting or being associated with youth culture**. They should not feature or portray real or fictitious characters who are likely to appeal particularly to people under 18. People shown using e-cigarettes or playing a significant role should not be shown behaving in an adolescent or juvenile manner.	6	‘The six macaron colored [product] series comes with extremely youthful coolness’
22.10 People shown using e-cigarettes or playing a significant role **must neither be, nor seem to be, under 25**. People under 25 may be shown in an incidental role but must be obviously not using e-cigarettes.	6	Photos of young adults holding ECs, wearing on-trend colourful clothes and makeup.
22.11 Marketing communications **must not be directed at people under 18 through the selection of media or the context** in which they appear. No medium should be used to advertise e-cigarettes if more than 25% of its audience is under 18 years of age.	5	Use of ‘memes’, links to social media platforms like Instagram, blog articles about ECs and unrelated topics (eg, music).
22.6 Marketers **must not use health professionals** to endorse electronic cigarettes.	4	Quotes from an addiction researcher and a professor of medicine to support the claim that ECs are less harmful than tobacco.
22.3 Marketing communications **must contain nothing which promotes the use of a tobacco product or shows the use of a tobacco product in a positive light**. This rule is not intended to prevent cigarette-like products being shown.	1	‘Cigarette-like satisfaction’
22.7 Marketing communications **must state clearly if the product contains nicotine**. They may include factual information about other product ingredients.	1	No mention of nicotine content.
22.4 Marketing communications **must make clear that the product is an e-cigarette** and not a tobacco product.	0	*None identified.*
22.2 Marketing communications **must contain nothing which** promotes any design, imagery or logo style that **might reasonably be associated in the audience’s mind with a tobacco brand**.	0	*None identified.*

* Smoking cessation claims are also considered to be medicinal claims.^28^

ECs, electronic cigarettes; MHRA, Medicines and Healthcare products Regulatory Agency.

Following data collection, we subsequently coded whether the claims that were extracted from the websites were factual rather than promotional, in line with code 22.12 (‘Factual claims about products are permitted on marketers’ own websites’). Additional guidance on code 22.12^29^ specifies that it prohibits health claims (eg, that ECs are safer or healthier than tobacco) and smoking reduction or cessation claims (which are also prohibited by code 22.5). We identified both types of claims on EC brand websites (eg, ‘vaping has no link to cancer and doesn’t have the harmful toxins associated with tobacco’ and ‘make vaping more accessible for people that want to give up smoking’). We also identified instances of descriptive language (eg, ‘Delectable flavors. Delightful experiences’), promotional marketing (eg, ‘Get 5% off your first order’) and testimonials (eg, ‘my family members welcomed the switch to [brand]’), which may violate code 22.12.^20 29^

## Discussion

These findings offer an overview of the claims which people may be exposed to when browsing popular EC brand websites in England in 2022. We identified several claims which may appeal to youth and non-smokers, some of which are not explicitly regulated by the CAP codes. Overall, compliance with CAP regulations was low. We suggest that ensuring explicit phrasing of certain codes may facilitate enforcement, and that updates to align with recent evidence may help address the issue of EC uptake by youth and non-smokers.

Our assessment of CAP code compliance revealed that most brands violated the rules regarding appeal to youth and non-smokers. Many websites included contents which may encourage youth and non-smokers to use ECs, pictures of models who look younger than 25 years using ECs and types of media which target youth (codes 22.8–22.11). It should be noted that some of these potential CAP code violations were implicit. For instance, claims about EC products being ‘beginner friendly’ may target smokers as much as non-smokers, and the exact age of a model cannot be determined from a picture. Nonetheless, depictions of young-looking people using ECs can promote normative beliefs about their use; in turn, perceived norms can influence risk perceptions and behaviour, especially among adolescents.^30^,^32^ The use of norm messaging was noted by a previous content analysis of EC advertising in England.^25^

We also identified health-related claims which may violate CAP codes. Most brands featured claims about EC use for smoking cessation, whereas the CAP prohibits smoking cessation claims concerning medically unlicensed EC products (code 22.5).^28^ While evidence suggests that nicotine-containing ECs may be more effective than nicotine replacement therapy as a smoking cessation aid, especially when used daily,^3 33^ some brands made claims such as ‘These devices will help you quit smoking’ without providing further evidence. Some of the identified health claims, such as reduced harm compared with tobacco and presenting ECs as risk-free, are also promotional according to CAP guidelines.^20^ To make health claims about an EC product, evidence specific to that product or range is required.^28^ Further consideration should be given as to how CAP regulations can reflect the evidence that ECs are harmful, but less harmful than combustible cigarettes,^1 2 4 5^ without implying regulatory approval or assurance for specific products. Consideration should also be given to claims about EC use for smoking cessation, which, while currently prohibited, may appeal to adult smokers more than to youth or non-smokers.

Additionally, we identified marketing claims which might appeal to youth and are not explicitly regulated by CAP codes. Claims about social enhancement are associated with increased susceptibility to use ECs among non-smoking youth,^34^ and claims linking products with personal identity may be particularly appealing during the transitional time of adolescence and young adulthood. Further, most EC websites advertised many flavours. While flavours may be associated with improved cessation rates in adult smokers,^35^ their promotion may be associated with likelihood of uptake and youth interest in trying ECs.^36 37^ We identified some claims concerning collaborations with artists, events and venues, which are promotional and may target a younger audience. Financial promotion claims featured on all websites, including those which did not offer direct retail. Such promotional claims, including sweepstake offers and giveaways, have been found to increase use interest among youth.^20 38^ Weight management is another example of marketing claims which can appeal to youth and non-smokers, such as claims about enjoying sweet flavours with no calories. Susceptibility to weight-related body image issues is high during adolescence, and weight-loss intentions are associated with EC use in youth.^39 40^

Our findings highlight that several CAP codes lack clear operationalisation and are too vague, which makes it difficult to determine objectively whether certain marketing content violates regulations. For instance, code 22.12 stipulates that marketers should only use factual claims^19^ but assessing whether a claim is factual or promotional is complicated even with the CAP’s additional guidance.^29^ We excluded CAP code 22.1 altogether because it was too challenging to objectively assess whether marketing content was ‘socially responsible’.^19^ To enhance compliance assessment and regulation enforcement, we suggest providing explicit definitions for the vague codes, as recommended by Stead *et al*.^25^ Efficient regulation of the marketing contents used by popular manufacturers on their own websites, which provide insights into the contents they use across platforms, may help manage broader exposure to EC marketing and, indirectly, the uptake of EC products by youth and non-smokers.

### Limitations

Some limitations should be considered when interpreting these findings. The EC market is diverse and fast changing; therefore, top brand data may quickly become outdated. Indeed, it does not reflect the recent surge in popularity of disposable EC products, particularly among youth, which has brought new brands to the top of the market in England.^6 41^ Furthermore, brand websites may not reflect the exact marketing claims potential users might encounter at the point of sale or through other channels like social media, although they do offer an extensive and frequently updated overview of the brands’ marketing strategies. While our frequency data reflect whether a claim appears on a website, they do not reflect how prominently it is placed or how frequently it is repeated on each website. For instance, we found that all websites featured at least one nicotine statement, but that does not mean that each nicotine-containing product was accompanied by a nicotine statement. Further research considering the frequency and visibility of each claim is needed. Because some CAP codes were open to subjective interpretation, we may have coded violations where regulatory bodies would not have. Finally, we acknowledge that while it is important to reduce EC marketing that targets youth, marketing content that appears to target adults may also be appealing to youth, as with many forms of tobacco marketing.

## Conclusions

Our findings highlight non-compliance with the advertising regulations established by the CAP on the websites of popular EC brands in England. The CAP codes aim to limit the impact of EC marketing claims on youth and non-smokers, yet codes concerning appeal to youth and non-smokers were some of the most violated, second only to medicinal claims. We also identified marketing elements which may be considered promotional, including the financial offers and claims relating to sensory experiences identified across all websites, or claims concerning collaborations with artists, venues and events. However, it is at times unclear which of these claims are prohibited by the CAP codes. Ensuring further clarification of existing regulations might facilitate their assessment and enforcement. The CAP codes could also be updated to reflect recent evidence concerning the relative health risks of ECs compared with tobacco products and EC use for smoking cessation.

## Supplementary material

10.1136/tc-2023-057934 online supplemental file 1

## Data Availability

Data are available upon reasonable request.
