# Supplementary material for: Marketing claims on the websites of leading e-cigarette brands in England
Source: Tob Control. 2023 Jul 4;34(1):e057934. doi: 10.1136/tc-2023-057934 (PMC11877088; doi:10.1136/tc-2023-057934)
Supplement: online supplemental file 1 [file tc-34-1-s001.pdf]

APPENDIX

Appendix 1. Codebook.

Code	Notes	Coding Options
**Section 1: Website Demographics**		
Website	Website data was collected from (URL)	[string]
Date1	Date the website was accessed - coder 1	[string]
Date2	Date the website was accessed - coder 2	[string]
Date3	Date the website was accessed - consensus coder	[string]
WebsiteType	Whether the website is UK-specific (i.e., ends in .uk, gb/en, en/gb) or non-UK specific (i.e., ends in .com)	1= UK specific website, 2= non-UK specific website
Brand	Brand/manufacturer website	1=Smok; 2=JUUL; 3=Blu; 4= 88 vape; 5=Vype; 6=Aspire; 7=Voopoo; 8=Logic; 9=VIP; 10=Vaporesso; 11= Innokin; 12=OK Vape
DOB	Does the website ask you about your age or DOB before entering the website?	0= No, 1= Yes
**Section 2: Product Types**		
TankPen	Presence of open/tank pen devices on website	1= Yes; 0= No
TankBox	Presence of open/tank box devices on website	1= Yes; 0 = No
Cigalike	Presence of 'cigalike' devices on website	1=Yes; 0 = No
PodDev	Presence of pod/cartridge devices on website	1= Yes; 0 = No
PodDev2	If PodDev=1: were the pod/cartridge devices shown on websites re-fillable or disposable?	1= Re-fillable; 2= Disposable; 3= Re-fillable and Disposable; 4= I'm not sure
Eliquids	Presence of E-liquid products on website (excluding disposable products)	1= Yes; 0 =No
Eliquids2	If Eliquids=1: Is it on a bottle or pod?	1=Bottle, 2= Pod, 3=Bottle and Pod,

		0=No; 99=Not applicable
**Section 3: Marketing Claims**		
**Flavour**	Does the website provide information about availability of different flavours?	0=No; 1=Yes
Flavour_txt	If Yes to Flavour (Flavour=1): was this information depicted in text?	0= No; 1=Yes; 99= Not applicable
Flavour_txt2	If Yes to Flavour_txt (Flavour_txt=1): Please include the exact phrase/sentence referring to this information. Please include up to five examples.	[string]
Flavour_img	If Yes to Flavour (Flavour=1): was this information depicted in an image?	0= No; 1=Yes; 99= Not applicable
Flavour_vid	If Yes to Flavour (Flavour=1): was this information depicted in a video?	0= No; 1=Yes; 99= Not applicable
**Colour**	Does the website include information about availability of different colours?	0= No; 1= Yes
Colour_txt	If Yes to Colour (Colour=1): was this information depicted in text?	0= No; 1=Yes; 99= Not applicable
Colour_txt2	If Yes to Colour_txt (Colour_txt=1): Please include the exact phrase/sentence referring to this information. Please include up to five examples.	[string]
Colour_img	If Yes to Colour (Colour=1): was this information depicted in an image?	0= No; 1=Yes; 99= Not applicable
Colour_vid	If Yes to Colour (Colour=1): was this information depicted in a video?	0= No; 1=Yes; 99= Not applicable
**Nicsalt**	Does the website feature claims about use of nicotine salts technology or availability of nicotine salts products?	0=No; 1=Yes
Nicsalt_txt	If Yes to Nicsalt (Nicsalt=1): was the claim depicted in text?	0= No; 1=Yes; 99= Not applicable
Nicsalt_txt2	If Yes to Nicsalt_txt (Nicsalt_txt=1): Please include the exact phrase/sentence referring to this claim. If more than one claim was identified, please include up to 5 examples.	[string]
Nicsalt_img	If Yes to Nicsalt (Nicsalt=1): was the claim depicted in an image?	0= No; 1=Yes; 99= Not applicable
Nicsalt_vid	If Yes to Nicsalt (Nicsalt=1): was the claim depicted in a video?	0= No; 1=Yes; 99= Not applicable
**Nicstrength**	Does the website feature claims referring to nicotine strength of their products?	0=No; 1=Yes
Nicstrength_txt	If Yes to NicStrength (Nicstrength=1): was the claim depicted in text?	0= No; 1=Yes; 99= Not applicable
Nicstrength_txt2	If Yes to Nicstrength_txt (Nicstrength_txt=1): Please include the exact phrase/sentence referring to this claim. If more than one claim was identified, please include up to 5 examples.	[string]

Nicstrength_img	If Yes to NicStrength (Nicstrength=1): was the claim depicted in an image?	0= No; 1=Yes; 99= Not applicable
Nicstrength_vid	If Yes to NicStrength (Nicstrength=1): was the claim depicted in a video?	0= No; 1=Yes; 99= Not applicable
**EcigQuitSmok**	Does the website include messaging relating to use of cigarettes as a cessation aid/way to help quit smoking cigarettes?	0= No; 1= Yes
EcigQuitSmok_txt	If Yes to EcigQuitSmok (EcigQuitSmok=1): was the claim depicted in text?	0= No; 1=Yes; 99= Not applicable
EcigQuitSmok_txt2	If Yes to EcigQuitSmok_txt (EcigQuitSmok_txt=1): Please include the exact phrase/sentence referring to this claim. If more than one claim was identified, please include up to 5 examples.	[string]
EcigQuitSmok_img	If Yes to EcigQuitSmok (EcigQuitSmok=1): was the claim depicted in an image?	0= No; 1=Yes; 99= Not applicable
EcigQuitSmok_vid	If Yes to EcigQuitSmok (EcigQuitSmok=1): was the claim depicted in a video?	0= No; 1=Yes; 99= Not applicable
**EcigSmokAlt**	Does the website feature messaging relating to the use of e-cigarettes as an alternative to smoking? (e.g., ‘created to be a satisfying alternative to smoking', ‘appealing alternatives to smoking’).	0= No; 1= Yes
EcigSmokAlt_txt	If Yes to EcigSmokAlt (EcigSmokAlt=1): was the claim depicted in text?	0= No; 1=Yes; 99= Not applicable
EcigSmokAlt_txt2	If Yes to EcigSmokAlt_txt (EcigSmokAlt_txt=1): Please include the exact phrase/sentence referring to this claim. If more than one claim was identified, please include up to 5 examples.	[string]
EcigSmokAlt _img	If Yes to EcigSmokAlt (EcigSmokAlt=1): was the claim depicted in an image?	0= No; 1=Yes; 99= Not applicable
EcigSmokAlt _vid	If Yes to EcigSmokAlt (EcigSmokAlt=1): was the claim depicted in a video?	0= No; 1=Yes; 99= Not applicable
**EcigAbsHarm**	Does the website feature claims referring to e-cigarettes as risk-free or harmless?	0= No; 1= Yes website
EcigAbsHarm_txt	If Yes to EcigAbsHarm (EcigSmokAlt=1): was the claim depicted in text?	0= No; 1=Yes; 99= Not applicable
EcigAbsHarm_txt2	If Yes to Ecig AbsHarm _txt (Ecig AbsHarm _txt=1): Please include the exact phrase/sentence referring to this claim. If more than one claim was identified, please include up to 5 examples.	[string]
EcigAbsHarm _img	If Yes to Ecig AbsHarm (Ecig AbsHarm =1): was the claim depicted in an image?	0= No; 1=Yes; 99= Not applicable
EcigAbsHarm _vid	If Yes to Ecig AbsHarm (Ecig AbsHarm t=1): was the claim depicted in a video?	0= No; 1=Yes; 99= Not applicable
**EcigHarmRed**	Does the website feature messaging referring to e-cigarettes as less harmful compared to smoking?	0 = No; 1=Yes
EcigHarmRed_txt	If Yes to EcigHarmRed (EcigHarmRed=1): was the message depicted in text?	0= No; 1=Yes; 99= Not applicable

EcigHarmRed_txt2	If Yes to EcigHarmRed_txt (EcigHarmRed_txt=1): Please include the exact phrase/sentence referring to this message. If more than one message was identified, please include up to 5 examples.	[string]
EcigHarmRed_img	If Yes to EcigHarmRed (EcigHarmRed=1): was the message depicted in an image?	0= No; 1=Yes; 99= Not applicable
EcigHarmRed_vid	If Yes to EcigHarmRed (EcigHarmRed=1): was the message depicted in a video?	0= No; 1=Yes; 99= Not applicable
**EcigSHSExp**	Does the website make claims relating to how products do not bother non-smokers or expose others to second hand smoke?	0=No; 1= Yes
EcigSHSExp_txt	If Yes to EcigSHSExp (EcigSHSExp= 1): was the claim depicted in text?	0= No; 1=Yes; 99= Not applicable
EcigSHSExp_txt2	If Yes to EcigSHSExp_txt (EcigSHSExp_txt=1): Please include the exact phrase/sentence referring to this claim. If more than one claim was identified, please include up to 5 examples.	[string]
EcigSHSExp_img	If Yes to EcigSHSExp (EcigSHSExp= 1): was the claim depicted in an image?	0= No; 1=Yes; 99= Not applicable
EcigSHSExp_vid	If Yes to EcigSHSExp (EcigSHSExp= 1): was the claim depicted in a video?	0= No; 1=Yes; 99= Not applicable
**EcigSensory**	Does the website make claims about sensory experiences associated with e-cigarettes? (e.g., ‘getting a nicotine hit without smell of tobacco’, ‘freedom from the mess of ash’)?	0= No; 1=Yes
EcigSensory_txt	If Yes to EcigSensory (EcigSensory =1): was the claim depicted in text?	0= No; 1=Yes; 99= Not applicable
EcigLSensory_txt2	If Yes to EcigSensory_txt (EcigSensory_txt=1): Please include the exact phrase/sentence referring to this claim. If more than one claim was identified, please include up to 5 examples.	[string]
EcigSensory_img	If Yes to EcigSensory (EcigSensory =1): was the claim depicted in an image?	0= No; 1=Yes; 99= Not applicable
EcigSensory_vid	If Yes to EcigSensory (EcigSensory =1): was the claim depicted in a video?	0= No; 1=Yes; 99= Not applicable
**EcigQuality**	Does the website make claims relating to the quality of products (e.g., ‘guaranteed high quality products’, ‘made in [x] country’)	0= No; 1= Yes
EcigQuality_txt	If Yes to EcigQuality (EcigQuality=1): was the claim depicted in text?	0= No; 1=Yes; 99= Not applicable
EcigQuality_txt2	If Yes to EcigQuality_txt (EcigQuality_txt=1): Please include the exact phrase/sentence referring to this claim. If more than one claim was identified, please include up to 5 examples.	[string]
EcigQuality_img	If Yes to EcigQuality (EcigQuality=1): was the claim depicted in an image?	0= No; 1=Yes; 99= Not applicable
EcigQuality_vid	If Yes to EcigQuality (EcigQuality=1): was the claim depicted in a video?	0= No; 1=Yes; 99= Not applicable

**EcigCustom**	Does the website make claims regarding the ability to personalise, customise or modifying their product devices?	0= No; 1= Yes
EcigCustom_txt	If Yes to EcigCustom (EcigCustom=1): was the claim depicted in text?	0= No; 1=Yes; 99= Not applicable
EcigCustom_txt2	If Yes to EcigCustom_txt (EcigCustom_txt=1): Please include the exact phrase/sentence referring to this claim. If more than one claim was identified, please include up to 5 examples.	[string]
EcigCustom_img	If Yes to EcigCustom (EcigCustom=1): was the claim depicted in an image?	0= No; 1=Yes; 99= Not applicable
EcigCustom_vid	If Yes to EcigCustom (EcigCustom=1): was the claim depicted in a video?	0= No; 1=Yes; 99= Not applicable
**EcigModern**	Does the website feature claims that refer their products as ‘modern’, ‘advanced’, ‘state of the art’, ‘hip’, ‘cool’, ‘cutting edge’, ‘futuristic’ or ‘innovative’?	0= No; 1= Yes
EcigModern_txt	If Yes to EcigModern (EcigModern=1): was the claim depicted in text?	0= No; 1=Yes; 99= Not applicable
EcigModern_txt2	If Yes to EcigModern_txt (EcigModern_txt=1): Please include the exact phrase/sentence referring to this claim. If more than one claim was identified, please include up to 5 examples.	[string]
EcigModern_img	If Yes to EcigModern (EcigModern=1): was the claim depicted in an image?	0= No; 1=Yes; 99= Not applicable
EcigModern_vid	If Yes to EcigModern (EcigModern=1): was the claim depicted in a video?	0= No; 1=Yes; 99= Not applicable
**EcigConvenience**	Does the website feature claims that refers to the convenience of use of their products?	0= No; 1= Yes
EcigConvenience_txt	If Yes to EcigConvenience (EcigConvenience=1): was the claim depicted in text?	0= No; 1=Yes; 99= Not applicable
EcigConvenience_txt2	If Yes to EcigConvenience_txt (EcigConvenience_txt=1): Please include the exact phrase/sentence referring to this claim. If more than one claim was identified, please include up to 5 examples.	[string]
EcigConvenience_img	If Yes to EcigConvenience (EcigConvenience=1): was the claim depicted in an image?	0= No; 1=Yes; 99= Not applicable
EcigConvenience_vid	If Yes to EcigConvenience (EcigConvenience=1): was the claim depicted in a video?	0= No; 1=Yes; 99= Not applicable
**EcigCost**	Does the website feature claims that refers to cheaper cost compared to tobacco products (e.g., cheaper than cigarettes, better monetary value than using tobacco)?	0=No; 1= Yes
EcigCost_txt	If Yes to EcigCost (EcigCost=1): was the claim depicted in text?	0= No; 1=Yes; 99= Not applicable
EcigCost_txt2	If Yes to EcigCost_txt (EcigCost_txt=1): Please include the exact phrase/sentence referring to this claim. If more than one claim was identified, please include up to 5 examples.	[string]

EcigCost_img	If Yes to EcigCost (EcigCost=1): was the claim depicted in an image?	0= No; 1=Yes; 99= Not applicable
EcigCost_vid	If Yes to EcigCost (EcigCost=1): was the claim depicted in a video?	0= No; 1=Yes; 99= Not applicable
**EcigPersIdentity**	Does the website feature claims associating their products with personal identity? (e.g., ‘You work hard for forge your own path, that’s what [brand X] stands for.’)	0= No; 1=Yes
EcigPersIdentity_txt	If Yes to EcigPersIdentity (EcigPersIdentity=1): was the claim depicted in text?	0= No; 1=Yes; 99= Not applicable
EcigPersIdentity_txt2	If Yes to EcigPersIdentity_txt (EcigPersIdentity_txt=1): Please include the exact phrase/sentence referring to this claim.	[string]
EcigPersIdentity_img	If Yes to EcigPersIdentity (EcigPersIdentity=1): was the claim depicted in an image?	0= No; 1=Yes; 99= Not applicable
EcigPersIdentity_vid	If Yes to EcigPersIdentity (EcigPersIdentity=1): was the claim depicted in a video?	0= No; 1=Yes; 99= Not applicable
**EcigSocialise**	Does the website feature claims referring to an increased ability to socialise (e.g., ability to form new friendships, ability to hang out with friends, increased opportunity to party or hang out with friends at bars)?	0= No; 1=Yes
EcigSocialise_txt	If Yes to EcigSocialise (EcigSocialise=1): was the claim depicted in text?	0= No; 1=Yes; 99= Not applicable
EcigSocialise_txt2	If Yes to EcigSocialise_txt (EcigSocialise_txt=1): Please include the exact phrase/sentence referring to this claim. If more than one claim was identified, please include up to 5 examples.	[string]
EcigSocialise_img	If Yes to EcigSocialise (EcigSocialise=1): was the claim depicted in an image?	0= No; 1=Yes; 99= Not applicable
EcigSocialise_vid	If Yes to EcigSocialise (EcigSocialise=1): was the claim depicted in a video?	0= No; 1=Yes; 99= Not applicable
**EcigCollab**	Does the website feature information regarding collaborations with other icons (e.g., celebrities, street artists, musicians, brands)?	0= No; 1=Yes
EcigCollab_txt	If Yes to EcigCollab (EcigCollab=1): was the claim depicted in text?	0= No; 1=Yes; 99= Not applicable
EcigCollab_txt2	If Yes to EcigCollab_txt (EcigCollab_txt=1): Please include the exact phrase/sentence referring to this claim. If more than one claim was identified, please include up to 5 examples.	[string]
EcigCollab_img	If Yes to EcigCollab (EcigCollab=1): was the claim depicted in an image?	0= No; 1=Yes; 99= Not applicable
EcigCollab_vid	If Yes to EcigCollab (EcigCollab=1): was the claim depicted in a video?	0= No; 1=Yes; 99= Not applicable
**EcigSustainability**	Does the website feature claims about their products being sustainable or environmentally friendly? (e.g., ‘adoption of 100% recyclable environmentally sustainable packaging’, ‘biodegradable packaging’, ‘sustainable packaging’)	0=no; 1= Yes

EcigSustainability_txt	If Yes to EcigSustainability (EcigSustainability=1): was the claim depicted in text?	0= No; 1=Yes; 99= Not applicable
EcigSustainability_txt2	If Yes to EcigSustainability_txt (EcigSustainability_txt=1): Please include the exact phrase/sentence referring to this claim. If more than one claim was identified, please include up to 5 examples.	[string]
EcigSustainability_img	If Yes to EcigSustainability (EcigSustainability=1): was the claim depicted in an image?	0= No; 1=Yes; 99= Not applicable
EcigSustainability_vid	If Yes to EcigSustainability (EcigSustainability=1): was the claim depicted in a video?	0= No; 1=Yes; 99= Not applicable
**EcigFireSafety**	Does the website feature claims that their e-cigarette products are fire-safe? (e.g., 'fire safe')	0= No; 1= Yes
EcigFireSafety_txt	If Yes to EcigFireSafety (EcigFireSafety=1): was the claim depicted in text?	0= No; 1=Yes; 99= Not applicable
EcigFireSafety_txt2	If Yes to EcigFireSafety_txt (EcigFireSafety_txt=1): Please include the exact phrase/sentence referring to this claim. If more than one claim was identified, please include up to 5 examples.	[string]
EcigFireSafety_img	If Yes to EcigFireSafety (EcigFireSafety=1): was the claim depicted in an image?	0= No; 1=Yes; 99= Not applicable
EcigFireSafety_vid	If Yes to EcigFireSafety (EcigFireSafety=1): was the claim depicted in a video?	0= No; 1=Yes; 99= Not applicable
**EcigVendorProm**	Does the website feature any messages referring to promotions or offers (e.g., 'buy 1 get 1 free', flash sales, bundle pack offers, sales)?	0=no; 1= Yes
EcigVendorProm_txt	If Yes to EcigVendorProm (EcigVendorProm=1): was the claim depicted in text?	0= No; 1=Yes; 99= Not applicable
EcigVendorProm_txt2	If Yes to EcigVendorProm_txt (EcigVendorProm_txt=1): Please include the exact phrase/sentence referring to this claim. If more than one claim was identified, please include up to 5 examples.	[string]
EcigVendorProm_img	If Yes to EcigVendorProm (EcigVendorProm=1): was the claim depicted in an image?	0= No; 1=Yes; 99= Not applicable
EcigVendorProm_vid	If Yes to EcigVendorProm (EcigVendorProm=1): was the claim depicted in a video?	0= No; 1=Yes; 2= Not applicable
**Section 4: CAP code compliance**		
**CAP1**	Do the ads on the website promote any design, imagery or logo style that might reasonably be associated in the audience's mind with a tobacco brand?	0=No; 1=Yes; 2= I'm not sure
CAP1_txt	If CAP1= 1: Please provide a sentence or two outlining the reason for your response.	[string]
**CAP2**	Do the ads on the website promote the use of a tobacco product or show the use of a tobacco product in a positive light?	0=No; 1=Yes; 2= I'm not sure
CAP2_txt	If CAP2= 1: Please provide a sentence or two outlining the reason for your response.	[string]

**CAP3**	Do the ads on the website make it clear that the product is an e-cigarette and not a tobacco product?	0=No; 1=Yes; 2= I'm not sure
CAP3_txt	If CAP3= 1: Please provide a sentence or two outlining the reason for your response.	[string]
**CAP4**	Do the ads on the website contain medicinal claims unless the product is authorised for those purposes by the MHRA? (E-cigarettes may be presented as an alternative to tobacco but must do nothing to undermine quitting tobacco use). Note: Claims that a product can 'cure', 'restore', 'prevent', 'avoid', 'fight' or 'heal' are likely to be considered as medicinal and advertisers should avoid making reference to them. A product which claims to treat or prevent disease would fall within the definition of a medicinal product. Claims to relieve symptoms, to cure, to provide a remedy or heal a specific disease/adverse condition would also be regarded as a medicinal claim.	0=No; 1=Yes; 2= I'm not sure
CAP4_txt	If CAP4= 1: Please provide a sentence or two outlining the reason for your response.	[string]
**CAP5**	Do the ads on the website use health professionals to endorse e-cigarettes?	0=No; 1=Yes; 2= I'm not sure
CAP5_txt	If CAP5= 1: Please provide a sentence or two outlining the reason for your response.	[string]
**CAP6**	Do the ads on the website clearly state if the product contains nicotine? (They may include factual information about other product ingredients).	0=No; 1=Yes; 2= I'm not sure
CAP6_txt	If CAP6= 1: Please provide a sentence or two outlining the reason for your response.	[string]
**CAP7**	Do the ads on the websites contain content which might encourage non-smokers or non-nicotine users to use e-cigarettes?	0=No; 1=Yes; 2= I'm not sure
CAP7_txt	If CAP7= 1: Please provide a sentence or two outlining the reason for your response.	[string]
**CAP8**	Are the ads on the website likely to appeal to people under 18, especially by reflecting or being associated with youth culture? (e.g., featuring or portraying real or fictitious characters who are likely to appeal to people under the age of 18).	0=No; 1=Yes; 2= I'm not sure
CAP8_txt	If CAP8= 1: Please provide a sentence or two outlining the reason for your response.	[string]
**CAP9**	Do the ads on the website show people using e-cigarettes or playing a significant role who are, or seem to be, under 25?	0=No; 1=Yes; 2= I'm not sure
CAP9_txt	If CAP9= 1: Please provide a sentence or two outlining the reason for your response.	[string]
**CAP10**	Are the ads on the website directed at people under 18 through the selection of media or the context in which they appear? (No medium should be used to advertise e-cigarettes if more than 25% of its audience is under 18 years of age.)	0=No; 1=Yes; 2= I'm not sure

CAP10_txt	If CAP10= 1: Please provide a sentence or two outlining the reason for your response.	[string]
